# Disability-Adjusted Life Years for the COVID-19 Pandemic in the Mexican Population

**DOI:** 10.3389/fpubh.2021.686700

**Published:** 2021-08-17

**Authors:** Guillermo Salinas-Escudero, Filiberto Toledano-Toledano, Carmen García-Peña, Lorena Parra-Rodríguez, Víctor Granados-García, María Fernanda Carrillo-Vega

**Affiliations:** ^1^Hospital Infantil de México Federico Gómez, Centro de Estudios Económicos y Sociales en Salud, Ciudad de México, Mexico; ^2^Hospital Infantil de México Federico Gómez, Unidad de Investigación de Medicina Basada en Evidencia, Ciudad de México, Mexico; ^3^Instituto Nacional de Geriatría, Dirección de Investigación, Ciudad de México, Mexico; ^4^Instituto Mexicano del Seguro Social, Centro Médico Nacional Siglo XXI, Unidad de Investigación en Epidemiología y Servicios de Salud, Área de Envejecimiento, Ciudad de México, Mexico

**Keywords:** COVID-19, SARS-CoV-2, global burden of disease, México, DALYs, YLLs, YLDs

## Abstract

Mexico is one of the countries most affected by the COVID-19 disease. Although there is vast information on the disease, there still are unknown data on the societal and economic cost of the pandemic. To estimate this impact, the disability-adjusted life years (DALYs) can be a useful tool.

**Objective:** To assess the DALYs due to COVID-19 in Mexico.

**Methods:** We used the data released by the Mexican Ministry of Health to estimate the DALYs by the sum of the years of life lived with disability (YLDs) and the years of life lost (YLLs).

**Results:** A total of 1,152,885 confirmed cases and 324,570 suspected cases of COVID-19 have been registered. Half of the cases were men, with a median age of 43.4 ± 16.9 years. About 8.3% died. A total of 39,202 YLDs were attributable to COVID-19. The total YLLs caused by COVID-19 were 2,126,222. A total of 2,165,424.5 DALYs for COVID-19 were estimated. The total DALYs were the highest in people between 50 and 59 years. The DALYs for each COVID-19 case were the highest in individuals between 60 and 79 years.

**Conclusion:** The DALYs generated by the COVID-19 represent a more significant disease burden than that reported for other causes, such as the 2009 H1N1 influenza pandemic. Although it impacts all age groups in terms of disability, the most affected group are people over 50 years of age, whose risk of death is higher.

## Introduction

The novel coronavirus is spreading worldwide, causing diverse adverse consequences (1–7). Numbers continuously change, and currently, most countries face second and third waves, with a larger number of cases burdening of the health systems (8–13). Over 85 million individuals have been infected, with ~2 million deaths (14). Among the WHO regions, the American continent ranks first in the number of infections (43% of all accumulated cases) and deaths (47% of all deaths) (14). The United States and Brazil are in leading in these numbers with increasing rates of infection and mortality. Other countries in the continent have a high toll, such as Colombia and Mexico (14).

There is a thorough epidemiological characterization of the disease. In particular, sex (men) and old age have been consistently reported as risk factors for COVID-19 (15, 16). The Latin American region has shown to be especially vulnerable to the morbid consequences of this pandemic due to the high prevalence of chronic diseases (e.g., diabetes and hypertension) and obesity that have been associated with adverse outcomes of younger individuals (17–21). Moreover, inequality stakes. For example, there is evidence that, in Mexico, living in municipalities with higher marginalization and being indigenous is associated with higher transmission rates and an ominous course of the disease for those who develop it (22, 23).

Despite efforts to better understand the disease behavior, there are still unknown societal and economic costs. Disability-adjusted life years (DALYs) are a metric quantifying and combining premature death and disability resulting from the disease to estimate its impact on the population (24). DALYs represent a tool to determine both the burden of disease and the intervention effects such as mass vaccination. Estimates of DALYs from Italy, Korea, and Japan have been released. However, those for Latin America, and specifically for Mexico (24–26), are missing. Thus, the present study aimed to assess the DALYs due to COVID-19 in Mexico.

## Methods

### Study Area

Mexico has a population of 127.79 million people, out of which 51% are women. Of the total, 34.5% are people below 20 years, 54.2% are people between 20 and 59 years, and 11.3% are people aged 60 years and over (27). About 74% of the total is affiliated with one or more public health services (28). The life expectancy at birth in 2020 was 78.11 years for women and 72.37 years for men. The per capita gross product is US$19,162 and the health expenditure is US$1,154 (29).

### Methods

We used the data released by the Mexican Ministry of Health (30), whose characteristics have been previously described (17, 18, 30). In brief, the database includes information on negative, confirmed, and suspected COVID-19 cases. Data on demographic characteristics (sex, age, nationality, residence place, and migratory status) and risk factors for severe COVID-19 (smoking and pregnancy) were registered. Information on morbidity, type of medical attention received (outpatient management or hospitalization), the medical unit where attended (public or private), dates when individuals developed symptoms, dates when they were admitted to the hospital and died is available. No information on evolution can be explored using this dataset.

Although the first case in Mexico was reported on February 27, 2020, we assumed that symptoms began 5 days before the individual search for medical care, so all the cases that reported the start of symptoms on February 22, 2020 were included. The inclusion date for counting cases in the present analysis was December 4, 2020. All COVID-19 cases confirmed with real-time reverse transcription PCR (RT-PCR) assay, independently of the signs and symptoms (31), clinical association, or experts committee were included (32–34).

Suspected cases represent a substantial proportion of the dataset. These cases are defined as the following: a person with acute onset of fever and cough or acute onset of any three or more of the following signs or symptoms: fever, cough, general weakness/fatigue, headache, myalgia, sore throat, coryza, dyspnea, anorexia/nausea/vomiting, diarrhea, and altered mental status; a person residing or working in a setting with a high risk of transmission of the virus, for example, closed residential settings and humanitarian settings, such as camp and camp-like settings for displaced persons, any time within the 14 days before symptom onset; a person residing in or travel to an area with community transmission anytime within the 14 days before symptom onset; a person working in a health setting, including within health facilities and within households, anytime within the 14 days before symptom onset; a patient with severe acute respiratory illness (SARI: acute respiratory infection with history of fever or measured fever of ≥38°C; and cough; with onset within the last 10 days; and who requires hospitalization); or an asymptomatic person not meeting epidemiologic criteria with a positive SARS-CoV-2 antigen-detecting rapid diagnostic test (Ag-RDT) (31). Based on the reasoning that suspected cases could underestimate the DALYs, we decided to include those reported as suspected in the period between September 1 and December 31, 2020. We considered that these cases were potentially positive COVID-19 as the result of the PCR test was still pending. The total suspected cases were adjusted by the positivity rate for COVID-19 corresponding to the 1st week of December 2020 (47.2%) (35). The cases reported as negative to the RT-PCR assay or those with insufficient information for its classification were excluded.

The DALYs were estimated by summing the years of life lived with disability (YLDs) and the years of life lost (YLLs), (36) as follows:

DALY = YLDs + YLLs

For this purpose, YLDs were estimated considering the status of the individual. For death, the period from the onset of symptoms to death date was counted as the time of disability, regardless of health care received (ambulatory or inpatient). The dataset does not include information on the disease remission date or discharge date for those alive. However, the average time from the onset of symptoms to seeking medical attention is reported. Based on this information, the time between seeking care and discharge was estimated by a linear extrapolation, assuming that the disease had the same time course in dead and surviving patients. Finally, a ponderation by type of health care received (outpatient or hospitalization) was conducted. This estimation was done as follows:

YLDs (death) = (Death date—seeking medical attention date in deaths) + (seeking medical attention date in deaths—Symptoms date in deaths)
YLDs (no deaths) = (Discharge date—seeking medical attention date) + (seeking medical attention date in no deaths—Symptoms date in no deaths)
where Discharge date = [(Death date—seeking medical attention date in deaths)/(seeking medical attention date in deaths—Symptoms date in deaths)] ^*^ [seeking medical attention date in no deaths—Symptoms date in no deaths]

Although long-term effects of COVID-19 have been described (37, 38), the dataset does not include data on post-COVID-19 signs and symptoms. Therefore, disability time was counted as the period of limitation and not that for sequels. A disability weight of one during the disease period was assumed. No reference reports this weight, so we took into account some elements to support this decision. First, for patients who needed to be hospitalized, who needed mechanical ventilation machine, and who were entered to ICU, we considered that the weight is very close to the unit due to the severity of the disease. For the outpatient cases, regardless of whether they were suspected or confirmed cases, the majority have to be in quarantine or total isolation, and as a result, the period of disability corresponds to those days in quarantine. Additionally, it was considered that those individuals who survived recovered their full health when the quarantine period ends.

The YLLs were obtained by subtracting the age of the person at death to life expectancy reported in the Mexican life tables, adjusted for sex and age. For this purpose, the life expectancy of the Mexican population adjusted by sex for 2019 was used (39). The final estimate of YLLs includes a ½-year adjustment to avoid an underestimation or overestimation in the parameter:

YLLs = Life expectancy—(Age of death−0.5)

Two scenarios were estimated for the YLLs. The first with raw values and the second using a 5% discount. For people over 106 years, the value discounted for those of 105 years was used.

Additionally, the per capita DALYs were stratified into age classes. To this end, the total DALYs by age group were divided by the people with COVID-19 in each group.

Finally, the rates for DALYs, YLLs, and YLDs per 100,000 inhabitants were calculated using the population officially reported for the country (40).

### Statistical Analysis

Descriptive analysis was performed. Continuous variables are presented as means and SDs, and categorical variables are expressed as frequencies and percentages. Comparisons between confirmed and suspected cases were estimated through the Mann–Whitney test for continuous variables and χ^2^ for categorical variables. The dataset was analyzed using the statistical package software STATA 14. Microsoft Excel 365 was used for the preparation of charts and graphs.

### Ethics

The submission of a formal protocol to the research and ethics committees is not required as the dataset is public and does not have personal data that allows the identification of individuals. However, the data analysis was registered with the National Institute of Geriatrics (registration number DI-PI-002/202).

## Results

Between February 22 and December 4, 2020, 1,152,885 confirmed and 324,570 suspected cases of COVID-19 have been registered in Mexico. Half of the cases were men, with a median age of 43.4 ± 16.9 years. About 20% of the sample was hospitalized, 15% was intubated, 8.5% was entered into the ICU, and 8.3% died. Although comparison was not the'primary objective of the present analysis, a higher proportion of confirmed cases was hospitalized, intubated, and entered the ICU compared with the suspected cases (*p* < 0.01). In the group of confirmed cases, there was higher mortality (9.4 vs. 4.2%, *p* < 0.01). The period between the symptoms starting from admission and the occurrence of death was longer in the confirmed cases (*p* < 0.01) (Table 1).

**Table 1:** General characteristics^a^.

	**Total**	**Confirmed cases**	**Suspected cases**	***p***
	***n* = 1,477,455**	***n* = 1,152,885**	***n* = 324,570**	
Sex (men)	745,053 (50.4)	584,976 (50.7)	160,077 (49.3)	
				<0.001^**^
Age (years)	43.4 ± 16.9	44.2 ± 16.8	40.7 ± 16.8	
				<0.001^*^
Hospitalized	286,996 (19.4)	246,006 (21.3)	40,990 (12.6)	<0.001^*^
Intubated	42,809 (15.0)	37,967 (15.5)	4,842 (11.8)	<0.001^*^
ICU	24,267 (8.5)	21,106 (8.6)	3,161 (7.7)	
				<0.001^*^
Dead	121,981 (8.26)	108,215 (9.4)	13,766 (4.2)	
				<0.001^**^
Days Syntom–Admission	4.199 ± 3.4	4.2 ± 3.3	4.1 ± 3.8	<0.001^**^
Days Syntom–Death	12.759 ± 9.1	13.2 ± 9.1	9.3 ± 8.8	<0.001^**^
Days Admission–Death	7.984 ± 8.4	8.4 ± 8.4	5.0 ± 7.9	<0.001^*^

a *Continuous variables are presented as Means ± Standard Deviation; categorical variables are presented as Frequencies (percentages)*.

* *X^2^ and Exact Fisher's test*.

** *Mann-Whitney test*.

It can be seen in Table 2 that the fatality rate increased with age, doubling in the 50–59 age group compared with the preceding group, and is close to 45% in the 80–89, decreasing by about three percentage points in the oldest age group (older than 90 years). Among the suspected cases, the fatality rate is lower, especially in the adjusted cases, in which the highest rate is 16% in the oldest age group.

**Table 2:** Fatality rate for COVID-19 by age group.

**Age group**	**Cases**	**%**	**Deads**	**%**	**Fatality rate**
**Confirmed cases**					
0–9	12,974	1.13	244	0.23	1.88
10–19 y	37,992	3.30	168	0.16	0.44
20–29 y	190,823	16.55	1,212	1.12	0.64
30–39 y	253,037	21.95	4,325	4.00	1.71
40–49 y	241,618	20.96	12,164	11.24	5.03
50–59 y	199,416	17.30	23,396	21.62	11.73
60–69 y	123,544	10.72	30,091	27.81	24.36
70–79 y	64,614	5.60	23,835	22.03	36.89
80–89 y	24,835	2.15	11,072	10.23	44.58
≥90 y	4,032	0.35	1,708	1.58	42.36
Total	1,152,885		108,215		
**Suspected cases (100%)**					
0–9	6,944	2.14	33	0.24	0.48
10–19 y	14,280	4.40	25	0.18	0.18
20–29 y	70,300	21.66	195	1.42	0.28
30–39 y	75,651	23.31	610	4.43	0.81
40–49 y	64,783	19.96	1,657	12.04	2.56
50–59 y	47,567	14.66	2,939	21.35	6.18
60–69 y	25,554	7.87	3,767	27.36	14.74
70–79 y	13,211	4.07	2,842	20.65	21.51
80–89 y	5,232	1.61	1,430	10.39	27.33
≥90 y	1,048	0.32	268	1.95	25.57
Total	324,570				
**Adjusted suspected cases (47.2%)**					
0–9	3,467	2.17	7	0.22	0.20
10–19	8,201	5.13	14	0.45	0.17
20–29	36,866	23.08	35	1.12	0.09
30–39	36,060	22.58	104	3.31	0.29
40–49	30,187	18.9	288	9.18	0.95
50–59	22,980	14.39	572	18.23	2.49
60–69	12,518	7.84	886	28.23	7.08
70–79	6,452	4.04	756	24.09	11.72
80–89	2,500	1.57	404	12.87	16.16
≥90 y	487	0.3	72	2.29	14.78
Total	159,718				

A total of 39,202 YLDs attributable to COVID-19, primarily determined by the confirmed cases, were estimated. The burden of YLDs was highest in those people at the age of most significant productivity (30–59 years). The total YLLs caused by COVID-19 was 2,126,222; the confirmed cases significantly contribute to this figure. The highest number of YLLs was observed between 30 and 79 years (Table 3A). It can be noted that the discount mainly affects the YLLs, that is, the discount rate minimizes the adverse health outcomes of the YLLs that are spread over time. On the other hand, the YLDs are assumed to occur during the period of illness (<1 year). It can be seen that about 1 million YLLs decreased when applying the discount rate (Table 3B).

**Table 3:** Years of life lived with disability (YLDs) and the years of life lost (YLLs) by age group.

	**Confirmed cases**			**Suspected cases**				**Global**
**Age group**	**Frequency**	**Mean**	**Subtotal**	**Frequency**	**Mean**	**Subtotal**	**Adjusted by positivity**	
**A. WITHOUT DISCOUNT**								
**Disability (Years)**								
0–9	12,974	0.02	281.99	3,467	0.02	55	26	308
10–19	37,992	0.03	1060.22	8,201	0.02	156	74	1,134
20–29	190,823	0.03	5756.39	36,866	0.02	790	373	6,129
30–39	253,037	0.03	7897.11	36,060	0.02	783	369	8,267
40–49	241,618	0.03	7970.24	30,187	0.02	691	326	8,296
50–59	199,416	0.03	6915.71	22,980	0.02	538	254	7,170
60–69	123,544	0.04	4444.85	12,518	0.02	304	144	4,589
70–79	64,614	0.04	2267.60	6,452	0.02	157	74	2,342
80–89	24,835	0.03	815.27	2,500	0.02	58	28	843
≥90 y	4,032	0.03	120.56	487	0.02	10	5	125
Total	1,152,885		37,529.94	3,138		57,194	26,996	2,126,222
**Life lost (Years)**								
0–9	244	73.34	17,894	7	71.85	503	237	18,132
10–19	168	60.59	10,180	14	61.51	861	406	10,586
20–29	1,212	50.20	60,846	35	49.15	1,720	812	61,658
30–39	4,325	41.29	178,562	104	40.72	4,235	1,999	180,561
40–49	12,164	32.35	393,457	288	32.09	9,241	4,362	397,818
50–59	23,396	24.34	569,429	572	24.72	14,141	6,675	576,104
60–69	30,091	17.25	519,083	886	17.11	15,159	7,155	526,238
70–79	23,835	11.28	268,925	756	11.06	8,358	3,945	272,870
80–89	11,072	6.75	74,690	404	6.71	2,712	1,280	75,970
≥90 y	1,708	3.61	6,162	72	3.65	263	124	6,286
Total	108,215		2,099,227	3,138		57,194	26,996	2,126,222
**B. WITH DISCOUNT**								
**Disability (Years)**								
0–9	12,974	0.02	282	3,467	0.02	55	26	308
10–19	37,992	0.03	1,060	8,201	0.02	156	74	1,134
20–29	190,823	0.03	5,756	36,866	0.02	790	373	6,129
30–39	253,037	0.03	7,897	36,060	0.02	783	369	8,267
40–49	241,618	0.03	7,970	30,187	0.02	691	326	8,296
50–59	199,416	0.03	6,916	22,980	0.02	538	254	7,170
60–69	123,544	0.04	4,445	12,518	0.02	304	144	4,589
70–79	64,614	0.04	2,268	6,452	0.02	157	74	2,342
80–89	24,835	0.03	815	2,500	0.02	58	28	843
≥90 y	4,032	0.03	121	487	0.02	10	5	125
Total	1,152,885		37,530	159,718		3,543	1,672	39,202
**Life lost (Years)**								
0–9	244	20.40	4,978	7	20.36	142	67	5,046
10–19	168	19.89	3,342	14	19.92	279	132	3,473
20–29	1,212	19.16	23,224	35	19.07	668	315	23,539
30–39	4,325	18.16	78,551	104	18.09	1,881	888	79,439
40–49	12,164	16.62	202,206	288	16.57	4,773	2,253	204,458
50–59	23,396	14.54	340,236	572	14.66	8,384	3,957	344,193
60–69	30,091	11.90	357,942	886	11.83	10,485	4,949	362,891
70–79	23,835	8.85	210,876	756	8.71	6,588	3,110	213,985
80–89	11,072	5.86	64,921	404	5.84	2,360	1,114	66,035
≥90 y	1,708	3.42	5,848	72	3.47	250	118	5,966
Total	108,215		1,292,123	3,138		35,809	16,902	1,309,025

A total of 2,165,424.5 DALYs for COVID-19 was estimated. When applying the discount rate, 1,384,227.4 DALYs resulted. The total DALYs were highest in the 50–59 years age group but were higher in the 60–69 years group when applying the discount rate. Almost two DALYs can be attributable to each COVID-19 case. It can be seen that each COVID-19 case between 60 and 79 years of age generates the most DALYs (Table 4).

**Table 4:** Total disability-adjusted life years (DALYs) by COVID-19 case.

**Age group**	**COVID cases and suspect cases adjusted**	**Premature dead**	**Premature dead discounted**	**Disability**	**Disability discounted**	**Total**	**Total discounted**	**Life of years lost by case**	**Life of years lost by confirmed case discounted**
0–9	12,977	18,131.6	5,045.7	307.8	307.8	18,439.4	5,353.5	1.42	0.41
10–19	37,999	10,586.0	3,473.4	1,134.0	1,134.0	11,720.0	4,607.4	0.31	0.12
20–29	190,840	61,657.9	23,538.9	6,129.1	6,129.1	67,787.0	29,668.0	0.36	0.16
30–39	253,086	180,560.7	79,439.3	8,266.6	8,266.6	188,827.3	87,705.9	0.75	0.35
40–49	241,754	397,818.2	204,458.4	8,296.4	8,296.4	406,114.6	212,754.8	1.68	0.88
50–59	199,686	576,104.0	344,192.9	7,169.5	7,169.5	583,273.5	351,362.5	2.92	1.76
60–69	123,962	526,238.3	362,890.5	4,588.6	4,588.6	530,826.8	367,479.1	4.28	2.96
70–79	64,971	272,869.7	213,985.5	2,341.9	2,341.9	275,211.6	216,327.4	4.24	3.33
80–89	25,026	75,970.5	66,035.0	842.9	842.9	76,813.4	66,877.9	3.07	2.67
≥90 y	4,066	6,285.5	5,965.7	125.4	125.4	6,410.9	6,091.1	1.58	1.50
Total	1,154,366	2,126,222	1,309,025	39,202	39,202	2,165,424.5	1,348,227.4	1.88	1.17

The general rate of YLLs, YLDs, and DALYs per 100,000 inhabitants was 1,663.8, 30.7, and 1,055, respectively. The highest rates of YLLs per 100,000 were observed in the 60–69 and the 70–79 years groups. The highest rate of YLDs per 100,000 was observed in the 50–69 years group. Finally, the highest rate of DALYs per 100,000 was observed in the 70–79 years group (Table 5).

**Table 5:** YLLs, YLDs, and DALYs per 100,000 inhabitants in México.

**Age group**	**Total population**	**YLLs per 100,000**	**YLDs per 100,000**	**DALYs per 100,000**
0–9	21,849,433	83.0	1.4	24.5
10–19	22,185,367	47.7	5.1	20.8
20–29	21,479,518	287.1	28.5	138.1
30–39	18,897,970	955.5	43.7	464.1
40–49	16,198,907	2,455.8	51.2	1,313.4
50–59	12,720,337	4,529.0	56.4	2,762.2
60–69	8,199,671	6,417.8	56.0	4,481.6
70–79	4,225,668	6,457.4	55.4	5,119.4
80–89	1,685,933	4,506.1	50.0	3,966.8
≥90 y	349,482	1,798.5	35.9	1,742.9
**Total**	**127,792,286**	**1,663.8**	**30.7**	**1,055.0**

## Discussion

The present study represents the first effort to understand the burden of COVID-19 in Mexico. According to our results, the total DALYs were the highest in people between 50 and 59 years, followed by individuals in the 60–69 years old group. The DALYs for each COVID-19 case were the highest in individuals between 60 and 79 years.

In line with official reports, the COVID-19 was the second cause of death in Mexico from January to August 2020, standing below cardiovascular disease and above diabetes, the two leading causes of death in the previous years (41). This information can sustain our results on mortality (YLLs) as the component that primarily determined the actual pandemic burden in Mexico, contributing 97.1% to the total DALYs. Similar results on the burden of the COVID-19 in other countries had been previously reported (24, 25).

A significant finding is that most YLLs were observed in individuals between 50 and 59. This information is similar to that previously reported about low and mid-income countries, where a significant fraction of the YLLs caused by the COVID-19 is from individuals dying at ages 55 or younger (42). During the AH1N1 pandemic of 2009, a similar behavior was observed; the younger population (20–59 years) determined the most YLLs (43). Notably, the'weight of YLLs for individuals between 20 and 59 years in our population was considerably higher (1,216,140.75 YLLs) in the COVID-19 pandemic than for the same age group during the 2009 pandemic (467,640 YLLs), even though the follow-up period in the present analysis was shorter.

In the present analysis, YLDs contributed less to total DALYs. This information is in harmony with other reports on the burden of the COVID-19 disease (24, 25) but not with reports on the DALYs generated by other communicable diseases (44, 45). This disagreement can be explained by the lack of information about the disability caused by the COVID-19. In this respect, it should be considered that some sequelae of the disease could appear long after having suffered it and for a prolonged period; consequently, we need to have more accurate data in this regard. The information generated on the sequelae and disability of COVID-19 will be beneficial as YLDs are considered an indispensable component for decision-making as they allow estimating the long-term effects of the disease.

Establishing information on the extended effects is of significant interest since it would allow setting the implications for both individuals and the health system in the future at a social and economic level. Nevertheless, it must be recognized that the information generated thus far had served as the basis for decision-making regarding COVID-19 vaccination campaigns. For example, it is well-known that the chronological age is not the condition that increases the risk of severe disease and death but the multimorbidity and frailty associated with a person. Thus, prioritizing vaccination in this group (46) could reduce the burden of COVID-19 and the high costs of its care.

It is essential to highlight that, in Mexico, as in other countries, the COVID-19 pandemic has strongly impacted the care of other diseases due to saturation at hospitals, the decrease in available personnel, and the redirection of resources to attend the pandemic emergency. The global production of medicines has been affected by social distancing and quarantines, decreasing medical treatments supply worldwide. Consequently, changes in the trend of the burden of other diseases will be observed in the following months, when complications from neglected diseases, including disability and death, appear more evident.

As a reflective exercise, we consider that the burden of the disease can also measure the effectiveness of public health policies and commitment of society to them. Up to this point in the pandemic, it is possible to say that there have been some contradictions in the official discourse of political and health leaders. The social commitment to adherence of basic norms of social distancing and hygiene and the use of face masks have not been as strong as desired, likely because of the lack of clear messages from policymakers. This lack of adherence has significantly determined the continuity in the virus transmission rate and the incidence of severe cases and mortality. Notwithstanding, as stated by the United Nations, the virus does not discriminate; but its impacts do (47). In this sense, it is necessary to reconsider that, in low and mid-economic counties, the effect of a total restriction in mobility also implies inequalities in access to resources, healthcare, education, social protection, and an exacerbation of discrimination, gender inequality, xenophobia, and domestic violence (48). Therefore, the pandemic response must come from different sectors so that the vaccination campaigns allow the transmission of the virus to cease to decrease the impact of YLDs and YLLs.

There are some limitations to this study. First, since the public health strategy of Mexico has not been applying massive detection tests, a significant percentage of asymptomatic and mild level cases could be under-registered. A slight impact on the YLLs would be expected because of these cases. Another factor to consider would be that COVID-19 deaths may not be correctly recorded, underestimating the actual lethality associated with COVID-19 and, therefore, the estimated YLLs.

Second, as mentioned above, information on the long-term effects of COVID-19 is not available in the present dataset, but the sequelae can lead to considerable underestimation of the total disease burden was not taken into consideration. That is why we decided to approach the phenomenon of disability through a coefficient of 1. Nevertheless, we suggest carefully analyzing the results on YLDs and consider that probabilities can change depending on other factors. For example, a coefficient <1 can be observed in those positive cases that, despite being in quarantine, carry out activities close to normal at home. Another consideration would be mild cases of illness that did not contact health providers and continued their normal activities outside the home, and asymptomatic cases that would not have sequelae. Despite these limitations, our results advanced in the phenomenon of disability secondary to COVID-19 and could give birth to new estimation strategies.

When interpreting our results, it should be considered that, by equating the duration of the disease in the cases that survived and those who died, we can overestimate the number of survivors. This assumption, however, corrects in some way the lack of data on the discharge of cases that did not die and was the approach that we consider the most appropriate to solve this limitation.

Finally, it is likely that when reading this study, the inclusion of suspected cases in the analysis will be questioned. In this regard, we suggest considering that several limitations in delivering laboratory test results are observed in Mexico. This fact represents a source of bias that tends to underestimate positive cases. That is why we tried to solve this limitation by applying the positivity rate to the suspected cases.

## Conclusions

The DALYs generated by the COVID-19 represent a more significant disease burden than that reported for other causes, such as the 2009 H1N1 influenza pandemic. Although it impacts all age groups in terms of disability, the most affected group are people over 50 years of age, whose risk of death is higher. The data shown in the present study, together with other previously published, should be taken into account to plan disease prevention and control strategies, including vaccination campaigns and the necessary social distancing and hygiene measures to prevent the spread of the virus and its new strains.

## Data Availability Statement

Publicly available datasets were analyzed in this study. This data can be found here: https://datos.gob.mx/busca/dataset/informacion-referente-a-casos-covid-19-en-mexico.

## Author Contributions

GS-E and MC-V: conceptualization. GS-E, MC-V, and FT-T: data curation and supervision. GS-E and LP-R: formal analysis. MC-V, GS-E, CG-P, and VG-G: investigation. MC-V, GS-E, CG-P, VG-G, and FT-T: methodology. GS-E, MC-V, and CG-P: writing—original draft. GS-E, MC-V, CG-P, VG-G, LP-R, and FT-T: writing—review & editing. All authors contributed to the article and approved the submitted version.

## Conflict of Interest

The authors declare that the research was conducted in the absence of any commercial or financial relationships that could be construed as a potential conflict of interest.

## Publisher's Note

All claims expressed in this article are solely those of the authors and do not necessarily represent those of their affiliated organizations, or those of the publisher, the editors and the reviewers. Any product that may be evaluated in this article, or claim that may be made by its manufacturer, is not guaranteed or endorsed by the publisher.
